# Sub-Nanosecond Lifetime Measurement Using the Recoil-Distance Method

**DOI:** 10.6028/jres.105.008

**Published:** 2000-02-01

**Authors:** Ching-Yen Wu

**Affiliations:** Nuclear Structure Research, Laboratory, University of Rochester, Rochester, NY 14627

**Keywords:** lifetime measurements, low-lying states, recoil-distance method

## Abstract

The electromagnetic properties of low-lying nuclear states are a sensitive probe of both collective and single-particle degrees of freedom in nuclear structure. The recoil-distance technique provides a very reliable, direct and precise method for measuring lifetimes of nuclear states with lifetimes ranging from less than one to several hundred picoseconds. This method complements the powerful, but complicated, heavy-ion induced Coulomb excitation technique for measuring electromagnetic properties. The recoil distance technique has been combined with heavy-ion induced Coulomb excitation to study a variety of problems. Examples discussed are: study of the two-phonon triplet in ^110^Pd, coupling of the β and γ degrees of freedom in ^182,184^W, highly deformed γ bands in ^165^Ho, octupole collectivity in ^96^Zr, and opposite parity states in ^153^Eu. Consistency between the Coulomb excitation results and the lifetime measurements confirms the reliability of the complex analysis often encountered in heavy-ion induced Coulomb excitation work.

## 1. Introduction

Understanding of the electromagnetic properties of the low-lying states in nuclei is important because it constitutes the cornerstone of collective models. Enhancement of the electromagnetic transition rate between states, relative to the single-particle unit or Weisskopf unit (W.u.), can only be interpreted as resulting from the coherent motion of nucleons in nuclei, which is a unique and interesting feature of a many-body quantum system. Therefore, there is no need to overemphasize the importance of measuring such quantities.

There are two ways to measure the electromagnetic properties of the low-lying states in nuclei. One is Coulomb excitation or electromagnetic excitation. The advantage of this method is that the electromagnetic matrix elements, which determine the transitions between states, are identical to the ones that determine the excitation process. Therefore, the cross sections for the excited states are directly related to the electromagnetic matrix elements of interest. Moreover, the excitation cross sections are sensitive to not only the transition matrix elements but also the phase and magnitude of static matrix elements attributed to the reorientation effect. The latter is a measure of the shapes of nuclei. The disadvantage is that the dependence of the excitation cross sections on these electromagnetic matrix elements is complicated if too many excited states are involved and sometimes is difficult to unscramble. Nevertheless, tremendous progress has been made in this field with the help of rapidly improving computing power and has resulted in many examples of interesting physics [1,2].

The other method is to directly measure the lifetime of the excited state, which determines directly the electromagnetic transition rate. However, the lifetimes for most low-lying collective states are on the order between <1 ps and several hundreds of picoseconds, which, in most cases, is beyond both the detector and instrument resolution. The recoil-distance method (RDM), first suggested and applied in the lifetime measurement by Alexander and his co-worker in the late 1960s [3], probably is the most accurate method to measure lifetimes in such a time scale.

## 2. Recoil-Distance Method

**Fig. 1:**
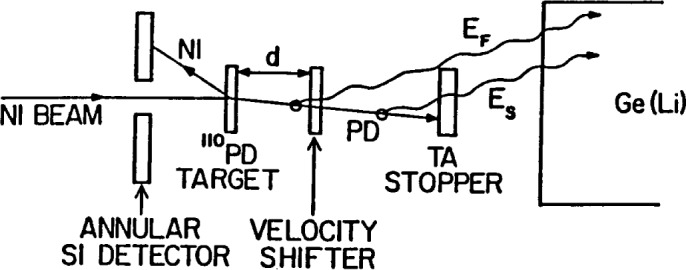
Schematic view for a typical setup of recoil-distance method.

A typical RDM setup, shown in Fig. 1, includes a foil placed downstream of the target, which is sufficiently thick to stop or slow-down the recoil particle significantly. In Rochester, the adopted foil is not thick enough to fully stop the recoil particles to avoid the Doppler-broadening lineshape problem. Since the excited states can decay either before or after the recoiling particles pass through the thick foil, the detected γ rays have two Doppler-shifted components for a given transition because of the velocity change. The intensity ratio between these two components thus is a measure of the electromagnetic transition rate. However, the lifetimes velocity vector is determined indirectly in our work by detecting the backscattered particles in coincidence with the detection of γ rays. The main function of the RDM apparatus is to control and calibrate the distance between the target and the foil and to achieve its parallelism.

The Rochester RDM apparatus was built in early 1980 [4] and the goal was to provide a complementary measurement to the Coulomb excitation program where the electromagnetic matrix elements determined from a complex analysis sometimes require an independent confirmation. The directly determined electromagnetic transition rate from the measured lifetime gives a certain confidence level to the results of Coulomb excitation experiments. The target-foil distance in Rochester RDM apparatus can be changed by a mechanical micrometer or by an “inchworm” which uses three piezo-electrical crystals to move a metal rod in fine ≈6 nm step over a range of 25 mm. Both push a parallel leafspring kinematic stage with the thick-foil frame mounted on it. The parallelism between the target and foil with an accuracy of 0.02° was achieved by comparing the position of the reflected laser beam at 3 m away from the target. The distance calibration was made by measuring the capacitance of the target-foil parallel system, which is inversely proportional to its separation at distances sufficiently small relative to the foil surface size.

In principle, one can determine the lifetime of an excited state with one single measurement at a givendistance. In practice, it is necessary to measure the decay *γ* rays at several distances to cover a wide time scale, to correct various feedings, and to minimize the systematic errors. The lifetimes were determined by fitting an exponential curve to the data after correcting the feedings and various attenuation to the γ-ray angular distribution, such as the finite size of Ge detector and the deorientation effect. A code, ORACLE [5], was developed originally to extract the lifetimes for a single cascade rotation band assuming the known external feedings. Two major modifications to ORACLE were made in Rochester to meet the need of more complicated analysis [6].

**Fig. 2:**
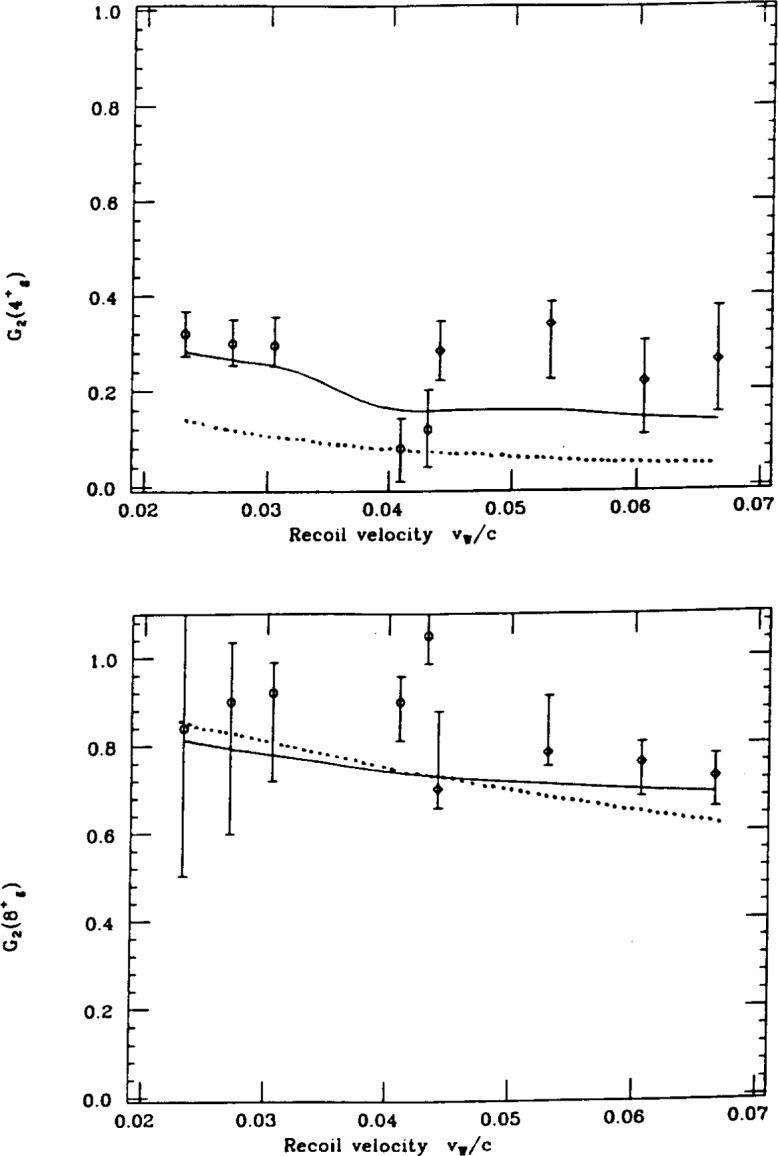
Comparison of the deorientation coefficient *G*_2_ between the experimental data and model prediction as a function of recoil velocity for the 4^+^ and 8^+^ states in ^182^W. Circles (diamond) are the data from the experiment with 232 MeV ^58^Ni (557 MeV ^136^Xe) as a projectile. Solid curve = two-state model. Dots = Abragam-Pound model.

One is to replace the Abragam-Pond [7] description of the deorientation effect by the “two-state” model [8] where the hyperfine field, generated by the atomic electrons at nucleus location, is assumed to consist of two parts according to the status of atomic electrons. In the fluctuating state, similar to the description of Abragam and Pond, the hyperfine field randomly orientates with a certain decay constant, caused by the de-excitation of the highly excited atomic configuration of the recoiling nucleus emerging from the target into the vacuum. In the static state, the hyperfine field is generated by the equilibrium atomic configuration. The agreement between our Coulomb excitation data and this model is reasonable [9,10] without any adjustment of the original parameters suggested in Ref. [8]. The sensitivity of the deorientation effect to the lifetime of the nuclear excited state is demonstrated in Fig. 2 here the comparison of the *G*_2_ coefficient between the experimental data and the model prediction is shown for the 4^+^ and 8^+^ states in ^182^W. The angular distribution is nearly washed out for the decay of the 4^+^ state with the mean lifetime about 100 ps.

## 3. Examples

As mentioned earlier, the Rochester RDM program complements our Coulomb excitation program. In the following examples, we are going to demonstrate how both measurements complement each other and the interesting physics that was identified and confirmed. In addition to cases discussed below, we also made lifetime measurements for ^94^Zr [11], ^148^Nd [12], and ^168^Er [13]. All the measurements mentioned here were performed using the Rochester RDM apparatus and the Rochester 16 MV tandem accelerator.

### 3.1 Two-Phonon Triplet in ^110^Pd

The vibrational structure dominating the low-lying states in ^110^Pd was the prevailing model used in the past because a closely spaced 0^+^, 2^+^, and 4^+^ triplet occurs at nearly twice the energy of the first 2^+^ state. Extensive Coulomb excitation work [14] shows that the second 0^+^ band is strongly deformed with *β*_2_ ≈ 0.35 compared to ≈ 0.26 for the ground state. This disparity in deformation among the triplet contradicts the anharmonic vibrator model often ascribed to nuclei in this mass region.

This stimulated the RDM measurement of the lifetimes for the excited states in ^110^Pd [6]. Comparison of the lifetimes measured by the RDM and derived from Coulomb excitation result is listed in Table 1. The over all agreement is reasonable despite the slightly deviation between two measurements for the $0_{2}^{+}$ and $2_{3}^{+}$ states. This agreement is significant for the second 0^+^ band and confirms its strongly deformed shape measured by Coulomb excitation.

### 3.2 Coupling Between the*β* and *γ* Degrees of Freedom in ^182,184^W

Nearly degenerate *β* and γ bands in W nuclei provide a favorable case for studying the coupling between these collective degrees of freedom. The pairing-plus-quadrupole model used by Kumar and Baranger [15] in solving the Bohr hamiltonian predicts a strong mixing between the *β* and γ vibrational degrees of freedom. A sharp decrease in the static quadrupole moment of the $2_{2}^{+}$ state in going from ^186^W to ^182,184^W seems to support this model prediction [16]. However, our Coulomb excitation results [17,18] do not substantiate this early claim. Moreover, our results also contradict the conclusion made by another Coulomb excitation work [19] that a sudden shape change occurs at spin 10 *ħ* of the ground-state band in ^182^W. An independent measurement is necessary to solve these contradictory claims.

Tables 2 and 3 list the comparison of the lifetimes measured by the RDM and derived from Coulomb excitation results for ^182,184^W, respectively. The lifetime measurement agrees reasonably well with our Coulomb excitation results and supports the claim that the interaction between the *β* and γ vibrational degrees of freedom is weak and the electromagnetic properties for the low-lying states are well correlated by a rotational mode of motion with a nonzero asymmetry of quadrupole deformation.

### 3.3 Highly Deformed *γ* band in ^165^Ho

The degeneracy of the γ band in even-even deformed nuclei is broken for odd-*A* deformed nuclei because of the nonzero K quantum number for the ground state. The deformed nucleus ^165^Ho has a ground state of spin 7/2^−^ and is an ideal case to study the identity of two well-known *γ* bands of K = 3/2^−^ and 11/2^−^ with bandheads at 515 keV and 688 keV respectively. An extensive Coulomb excitation study [20] shows that the intrinsic E2 moment for the K = 11/2^–^ band is about 10.5 eb which is about 40 % larger than the 7.4 eb for the ground-state band. The other band with K = 3/2^−^ has the intrinsic E2 moment about 8.6 eb. The finding of this highly deformed γ band was a surprise because the mixing with other deformed configurations is not expected at such a low excitation energy. Lifetime measurements for ^165^Ho by the RDM [21] in general supports this claim. So far, there is no viable explanation for this highly deformed γ band occurring at such a low excitation energy.

### 3.4 Octupole Collectivity in^96^Zr

Strong octupole correlations are expected to occur in ^96^Zr because of the coherent superposition of proton 2*p*_3/2_ →*g*_9/2_ and neutron 2*d*_5/2_ →1*h*_11/2_ particle-hole excitations. Two recent centroid-shift measurements [22,23] of the lifetime of the $3_{1}^{-}$ state at 1897.2 keV imply that the *B*(E3) value is about 65 W.u. to 69 W.u. This unusually enhanced octupole strength has been suggested to be inconsistent with simple harmonic vibrations and implies the possible existence of octupole shape instability and the breakdown of the random-phase approximation formalism for ^96^Zr [23].

**Fig. 3:**
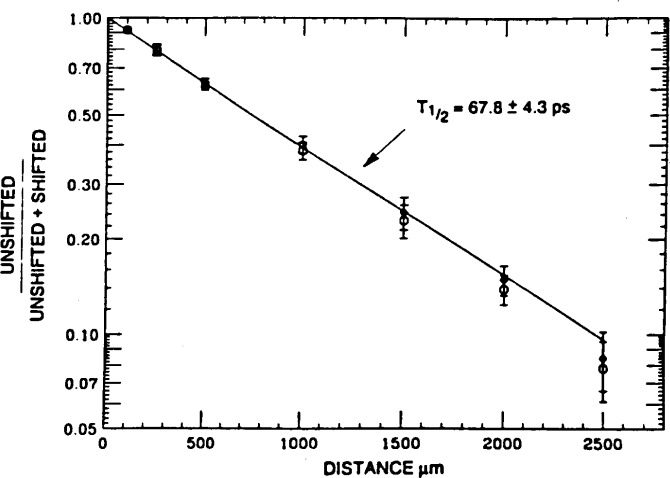
The intensity ratio of the unshifted peak to the sum of both the unshifted and shifted components of the ^96^ Zr 146.7 keV γ ray vs the distance between the target and thick foil. The circles represent the raw ratios while the diamonds correspond to the corrected data. The solid curve is the best fit to the corrected data.

The half-life of the $3_{1}^{-}$ state has been remeasured by the RDM and determined to be 67.8 ± 4.3 ps, as shown in Fig. 3, which implies *B*(E3) = 47.1 ± 4.7 W.u. [24]. This still is the most enhanced E3 transition observed in nuclei even though it is about 40 % weaker than those derived from the earlier centroid-shift measurements. Serious doubts are raised about their conclusion on the breakdown of a simple harmonic vibrator model for ^96^Zr.

The one-phonon octupole state in ^96^Zr has a relative low excitation and the most enhanced E3 transition observed in nuclei. This makes ^96^Zr an ideal case to study the two-phonon octupole states. The preliminary results from the recent Coulomb excitation experiment [2,25] indicate that the first 6^+^ state at 3483.4 keV, which was identified in the past as the possible candidate for the two-phonon octupole state because the characteristic E1 decay to the 5^−^ state at 3119.5 keV, accounts for only about 8 % of two-phonon harmonic strength. Instead, the second 6^+^ state at 3772.2 keV, which appears to be a member of the shape isomeric 0^+^ band, contains a substantial fraction of two-phonon strength. Detailed analysis still is in progress.

### 3.5 Opposite-Parity States in ^153^Eu

**Fig. 4:**
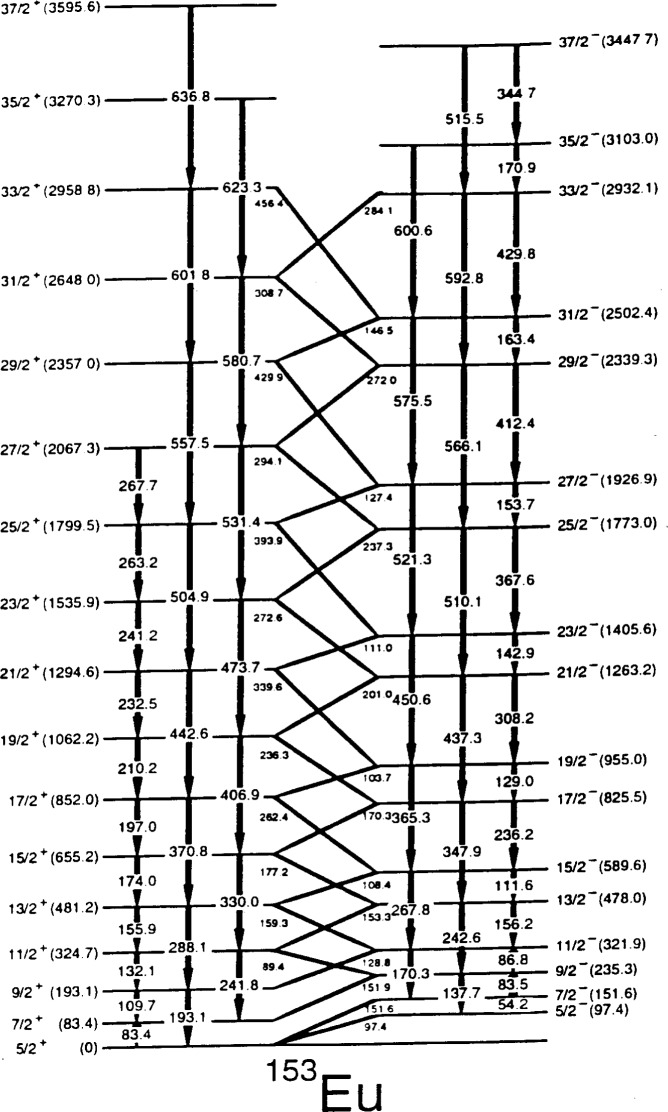
The partial level scheme for ^153^Eu.

One of the distinct characteristics of octupole deformation is the parity-doublet states in odd-*A* nuclei, where states with equal spin and opposite parity are nearly degenerate. Parity-doublet states have been observed in the light-actinide region, such as ^223^Th [26]. A recent study of ^153^Eu using the ^150^Nd(^7^Li,4n) reaction shows a pattern of parity-doublet states [27] as illustrated in the partial level scheme shown in Fig. 4. However, their model-dependent analysis based on the measured branching and mixing ratios, assuming both the K = 5/2^+^ and 5/2^−^ bands have the same intrinsic moment as that of the ground state, suggests that the *g*-factors differ by factors of three between the bands. This implies that the underlying single-particle structures are different from the assumed single intrinsic parity-mixing state.

**Fig. 5:**
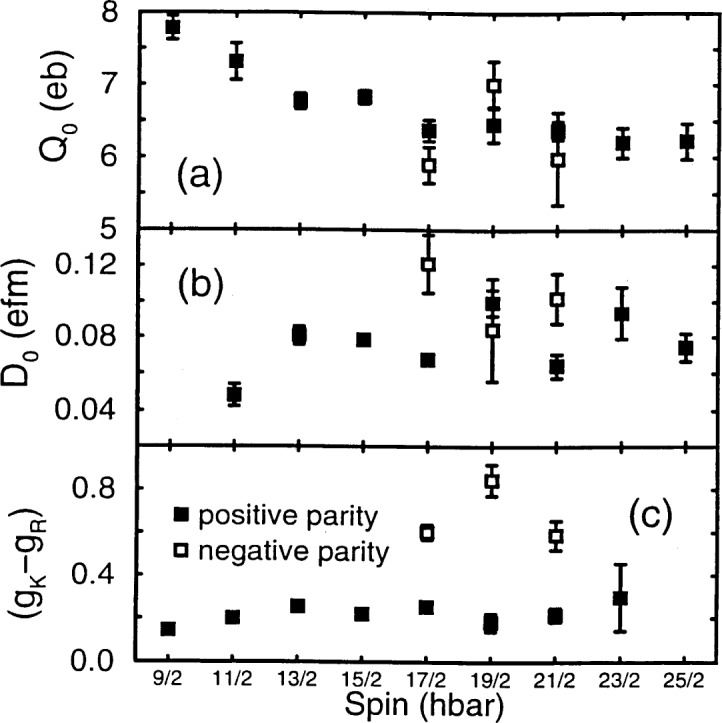
Moments and *g*-factors deduced from the lifetimes. The E2 moments derived from the I → (I–2) transitions are shown in (a), the E1 moments shown in (b), and the *g*-factors shown in (c).

Lifetime measurements for ^153^Eu [28] by the RDM adds additional constraints to the data and makes a model-independent analysis possible. The E2 and E1 moments derived from the lifetimes and the known branching and mixing ratios are shown as a function of spin in Fig. 5(a) and Fig. 5(b), respectively, and they are nearly the same for both K = 5/2^+^ and 5/2^–^ bands. In contrast, the derived *g*-factors differ substantially, as shown in Fig. 5(c). This work supports the conclusion, made by the early model-dependent analysis, that the bands have different intrinsic structure and therefore, are unlikely to be a pair of parity-doublet bands.

## 4. Summary

It has been demonstrated that the recoil-distance method is a very reliable and accurate tool to measure lifetimes between <1 ps and several hundreds of picosecond, which is the time scale of lifetimes for many low-lying collective states in nuclei. The RDM provides not only an independent measurement, which is an important complement to the Coulomb excitation technique, but also an important quantity in a complete set of measurable electromagnetic properties. Consistency between the Coulomb excitation results and lifetime measurements, shown in several cases presented here, prove that the complex analysis often encountered in heavy-ion induced Coulomb excitation is possible and valid.

## Figures and Tables

**Table 1:** Comparison of the lifetimes in picosecond between Coulomb excitation result and direct measurement by the RDM for ^110^Pd

Spin	Energy(keV)	Lifetimes (ps)	
		Coulomb excitation	RDM
$2_{1}^{+}$	373.8	63.2 ± 1.9	65.6 ± 2.5
$4_{1}^{+}$	920.8	$6.5_{-0.2}^{+0.4}$	5.9 ± 0.4
$6_{1}^{+}$	1573.8	$2.1_{-0.1}^{+0.2}$	2.0 ± 0.2
$2_{2}^{+}$	813.6	$26.8_{-1.3}^{+0.7}$	25.6 ± 1.2
$4_{2}^{+}$	1398.0	$7.8_{-0.6}^{+0.7}$	7.4 ± 0.8
$0_{2}^{+}$	946.7	$15.3_{-1.1}^{+0.6}$	11.4 ± 1.0
$2_{3}^{+}$	1214.5	$17.5_{-2.5}^{+1.4}$	13.1 ± 0.8
$4_{3}^{+}$	1718.5	$2.7_{-0.7}^{+0.5}$	3.2 ± 0.4

**Table 2:** Comparison of the lifetimes in picosecond between Coulomb excitation result and direct measurement by the RDM for ^182^W

Spin	Energy (keV)	Coul. ex. [18]	Lifetimes (ps) Coul. ex. [19]	RDM
$2_{1}^{+}$	100.1	2185 ± 90	1639 ± 180	
$4_{1}^{+}$	329.4	105 ± 5	88 ± 9	90 ± 4
$6_{1}^{+}$	680.5	11.7 ± 0.3	12.6 ± 0.3	11.9 ± 0.13
$8_{1}^{+}$	1144.5	3.03 ± 0.13	2.78 ± 0.29	2.90 ± 0.25
$10_{1}^{+}$	1712.1	1.23 ± 0.06	0.89 ± 0.12	1.10 ± 0.10

**Table 3:** Comparison of the lifetimes in picosecond between Coulomb excitation result and direct measurement by the RDM for ^184^W

Spin	Energy (keV)	Coul. ex. [18]	Lifetimes (ps) Coul. ex. [19]	RDM
$2_{1}^{+}$	111.2	1869 ± 79	1485 ± 158	
$4_{1}^{+}$	364.0	65 ± 3	81 ± 15	57 ± 7
$6_{1}^{+}$	748.3	8.3 ± 0.3	7.7 ± 0.8	7.5 ± 0.9
$8_{1}^{+}$	1251.9	2.14 ± 0.05	1.38 ± 0.18	1.98 ± 0.25
$10_{1}^{+}$	1860.5	0.82 ± 0.04	0.83 ± 0.09	0.95 ± 0.13
$2_{2}^{+}$	903.3	2.65 ± 0.12		3.3 ± 0.4
$4_{2}^{+}$	1133.8	3.32 ± 0.25		3.8 ± 0.5
$6_{2}^{+}$	1476.9	2.62 ± 0.13		2.9 ± 0.5
